# Perturbation of the circadian clock in chronic diseases involving organ fibrosis

**DOI:** 10.1172/JCI194018

**Published:** 2025-10-01

**Authors:** Atish Mukherji, Pierre-Louis Tharaux, David W. Ray, Thomas F. Baumert

**Affiliations:** 1 University of Strasbourg, Institute of Translational Medicine and Liver Diseases (ITM), Inserm UMR_S1110, Strasbourg, France.; 2 Université Paris Cité, Inserm, Paris Cardiovascular Centre–PARCC, Paris, France.; 3 National Institute for Health and Care Research (NIHR), Oxford Biomedical Research Centre, John Radcliffe Hospital, Oxford, United Kingdom.; 4 Oxford Centre for Diabetes, Endocrinology and Metabolism and; 5 Oxford Kavli Centre for Nanoscience Discovery, University of Oxford, Oxford, United Kingdom.; 6 Gastroenterology and Hepatology Service, Strasbourg University Hospitals, Strasbourg, France.; 7 Institut Universitaire de France, Paris, France.; 8 Institut Hospitalo-Universitaire (IHU) Strasbourg, Strasbourg, France.

## Abstract

Chronic organ disease is often complicated by fibrosis, the excessive accumulation of extracellular matrix, as a consequence of dysfunctional wound healing responses. Fibrosis progressively distorts tissue architecture and eventually leads to loss of organ function, accounting for up to 45% of deaths in developed countries. Moreover, fibrosis is a major risk factor for tumor development. The few approved therapies aimed at preventing or resolving fibrosis show limited efficacy and safety. One reason for the lack of efficient antifibrotic therapies is the fact that the cell circuits driving the disease biology are still only partially understood. The circadian clock is known to regulate the physiological functions of critical organs, including the liver, kidneys, and lungs. Several experimental and clinical studies have established that circadian disruption plays an important role in the development of chronic diseases across organs involving fibrosis. These include metabolic dysfunction–associated steatotic liver disease, chronic kidney disease, and chronic obstructive pulmonary disease. Here, we provide an overview of the circadian mechanisms that play critical roles in mediating physiological functions in the liver, kidneys, and lungs and whose deregulations could predispose toward development of chronic disease of these organs, leading to fibrosis. We also highlight the possible opportunities of chronotherapy for chronic diseases and discuss future perspectives.

## Introduction to the circadian clock: principles and components

**Figure 1:**
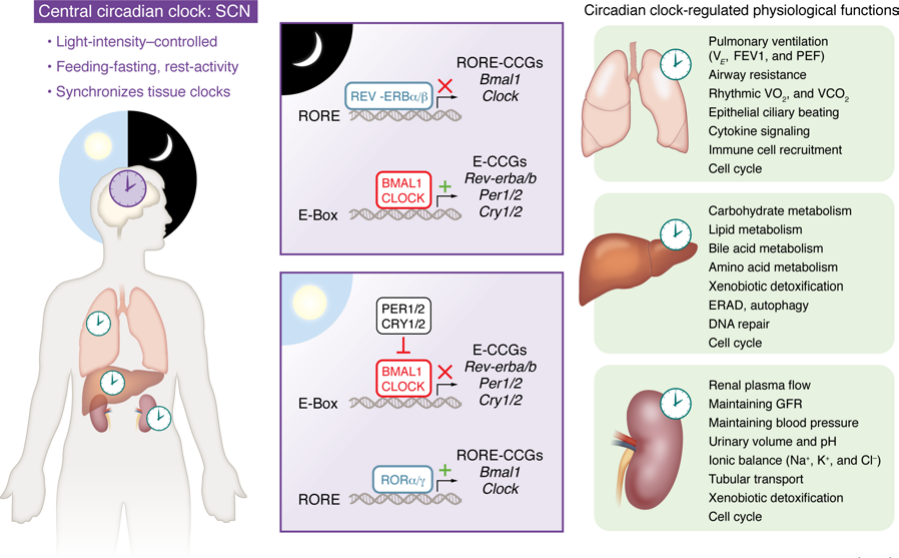
Circadian regulation of the cellular clock and physiological outputs. The central circadian clock in the suprachiasmatic nucleus (SCN) of the hypothalamus synchronizes peripheral clocks. Within each organ, every cell contains a circadian clock (CC) oscillator, based on a negative transcription-translation feedback loop, that drives expression of numerous clock-controlled genes (CCGs). The CC oscillators in different cell types are largely responsible for maintaining essential physiological functions. ERAD, endoplasmic reticulum–associated degradation; FEV1, forced expiratory volume in 1 second; GFR, glomerular filtration rate; PEF, peak expiratory flow; *V_E_*, pulmonary ventilation; *VO_2_*, oxygen consumed; *VCO_2_*, carbon dioxide exhaled.

Iconic solar worship sites in different continents suggest that humanity has been conscious of a world dominated by the daylight cycle throughout history. Most terrestrial life forms display biological rhythms with a period of approximately 24 hours, allowing them to prepare for daily variations of the light-dark cycle. This endogenous rhythm is known as a circadian rhythm, and it coordinates the physiology of organisms with the light cycle and behaviors, including feeding-fasting and sleep-wake cycles (1–6). Discovery of the first circadian gene in *Drosophila* initiated the molecular clock revolution (7). Mammalian circadian rhythms require periodic entrainment to remain synchronized with different “zeitgebers,” or time cues, e.g., the light-dark cycle (8–12). This synchronization process relies on light information from the retina passing to the hypothalamic suprachiasmatic nucleus, which is the site of the central circadian clock. This central clock then disseminates signals to the clocks in most cells of the body, ensuring they oscillate in phases (8–12) (Figure 1).

The cellular circadian clock (CC) runs as a transcription-translation feedback loop and is conserved across cell types (12–14) (Figure 1). The core of the CC oscillator is made up of two transcription factors: CLOCK (or its paralog NPAS2) and its partner BMAL1. In the rest phase (night for humans), the BMAL1-CLOCK complex is recruited to E-box motifs present in the regulatory regions of several genes, including the repressor families Period (*PER1*, *PER2*) and Cryptochrome (*CRY1*, *CRY2*) (12–14) (Figure 1). In the late rest phase, phosphorylated PER and CRY proteins heterodimerize to repress the activity of BMAL1-CLOCK, inhibiting their own expression (12–14). Additionally, BMAL1-CLOCK activates transcription of *REV-ERBa* and *REV-ERBb*, which in complex with HDAC3 are recruited to inhibit expression of RORE-containing genes, e.g., *BMAL1* and *CLOCK* (13–18). In active phase, RORα/γ activates *BMAL1* and *CLOCK* transcription, enabling the start of the next cycle (12–14, 19, 20) (Figure 1). Together, these mechanisms generate rounds of rhythmic transcription at different phases depending on the combination of DNA-binding elements (13–16). Depending on the organ/tissue, 10%–20% of the genome is transcribed rhythmically (21–27). The CC also influences splicing, mRNA polyadenylation, mRNA export, and translation (5, 13). Importantly, distinct circadian rhythms can persist in the absence of core clock gene expression through mechanisms that remain poorly understood. Additionally, peripheral clocks can be entrained by non-photic stimuli, notably food timing (28–32).

**Figure 2:**
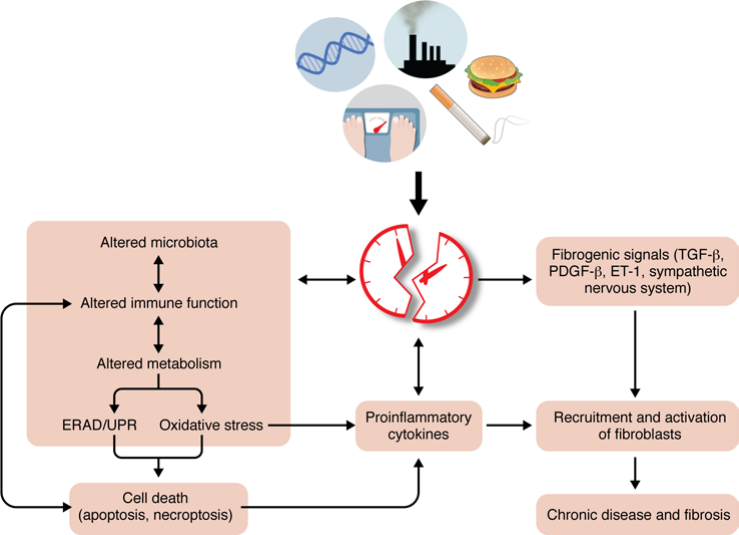
The perturbed “clock” as a candidate driver of chronic disease leading to fibrosis and organ failure. The model depicts a simplified global view of how various events (e.g., genetics, lifestyle, environment, habits) perturb the clock activity, which in turn alters multiple functions and pathways, leading to the development of chronic disease and fibrosis. The left side of the figure shows the bidirectional communication between the clock machinery and metabolism, microbiota signaling, and immune functions. This communication regulates cellular functions (such as ERAD/UPR, cytokine production, cell death, and oxidative stress) in a temporal manner, thereby maintaining homeostasis. As shown on the right side, perturbation of the clock disrupts metabolism, triggers activation of the immune system, and elevates stress responses, driving chronic diseases and fibrosis. Notably, mesenchyme-derived fibroblasts of different subtypes are known to harbor functional cellular clocks, which are disrupted in chronic disease, leading to deregulated production of key mediators and effectors of fibrosis. ERAD, endoplasmic reticulum–associated degradation; ET-1, endothelin-1; UPR, unfolded protein response.

Teleologically, zeitgebers are required to maintain the near-24-hour rhythms to optimize physiological adaptation (1–3, 12, 30). Circadian misalignment occurs when our internal rhythm is out of phase with the natural light-dark cycle (1–3, 30). Examples include jet lag and shift work, which are associated with increased risk of chronic diseases, including diabetes, metabolic syndrome, cardiovascular disorders, and renal disease, as well as fibrosis and cancer (23–27, 30, 33–35) (Figure 2).

Importantly, circadian rhythm disruption is also seen in early life (36, 37). Investigations in mouse models have shown that chronodisruption affects physiology, development, and growth in both pre- and postnatal life (36–39). Epidemiological studies indicate that circadian disruption during pregnancy via shift work is associated with adverse outcomes at birth (miscarriage, preterm delivery) and later in life (such as sleep disorder, bipolar disorder, susceptibility to infections, aging) (36–39).

Almost all chronic diseases induced by circadian perturbation, such as diabetes, metabolic syndrome, and inflammation, ultimately result in structural remodeling of their tissues through fibrosis. Thus, an understanding of how CC perturbation contributes to fibrosis during chronic metabolic or inflammatory disease is important in order to understand disease biology and therapeutic opportunities.

## Fibrosis: underlying common principles and molecular drivers

Fibrosis of solid organs is a major cause of morbidity and mortality worldwide. Advanced fibrosis ultimately leads to organ failure or cancer (40–44). Globally, fibrosis has emerged as a leading contributor to disease burden, affecting nearly 5,000 patients for every 100,000 cases (40–45). Despite large research and development efforts, antifibrotics for the kidney are lacking and have limited therapeutic efficacy in lungs and liver. Since the mechanisms underlying fibrosis have been extensively reviewed (40–55), here we provide a brief overview of common concepts across organs relevant to understanding their interplay with the CC.

Fibrosis is characterized by the accumulation of extracellular matrix (ECM) proteins in tissues, which distort their architecture and perturb their physiological functions (40–44). Fibrosis is not a disease per se but rather an outcome of the tissue’s reparative response to chronic injury. While the ability to repair wounds successfully is advantageous, excess matrix deposition in chronic disease states is detrimental (43, 46, 51, 55). This maladaptive accumulation of ECM can be triggered by multiple factors, e.g., viral or bacterial pathogens, high-fat diets, alcohol, smoking, drug toxicity, air pollutants, diabetes, and genetic mutations (43, 46, 51, 53–55) (Figure 2).

**Figure 3:**
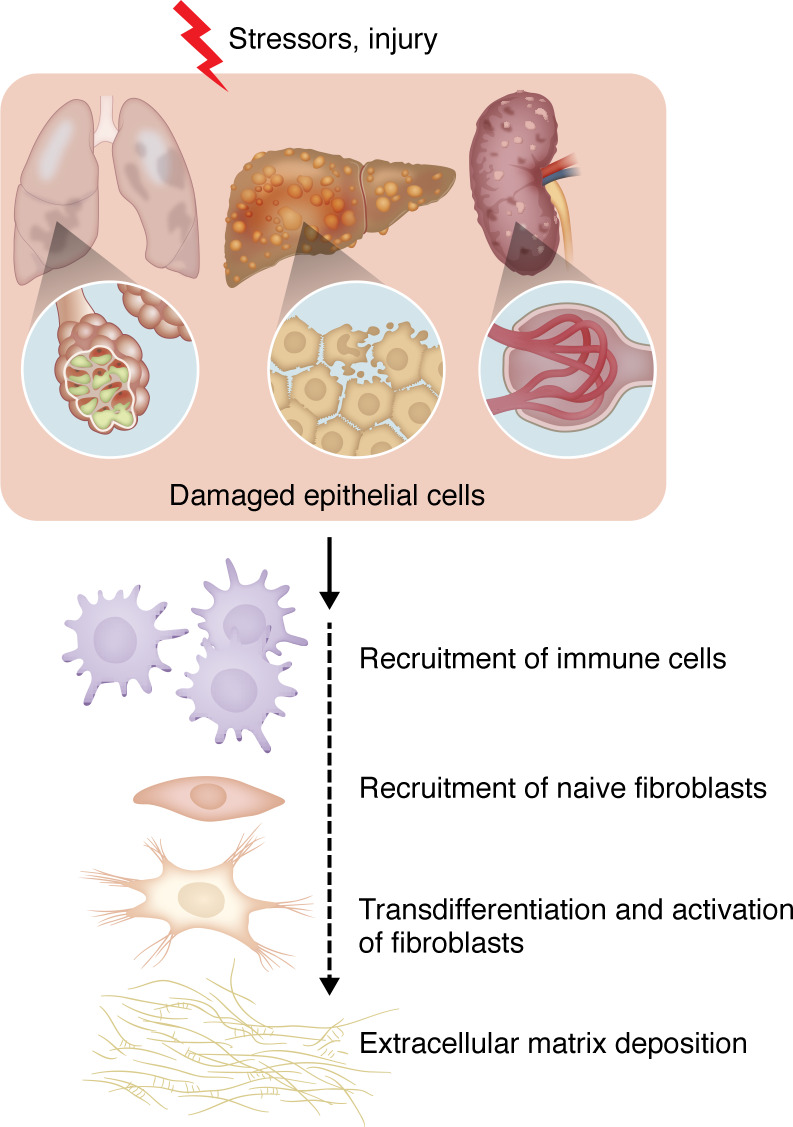
Common steps in organ fibrosis. Multiple metabolic and inflammatory stressors and injury damage epithelial cells in different organs, initiating the pathogenic cascade of fibrosis. In chronic disease, damaged epithelial cells raise an evolutionarily conserved protective response involving multiple cell types. Briefly, epithelial damage leads to attraction of immune cells, which secrete multiple factors to enable recruitment of naive fibroblasts of mesenchymal origin. Once recruited into this inflammatory milieu, fibroblasts are transdifferentiated and activated. With FGF, TGF-β, and PDGF-β as key mediators, fibroblasts produce and secrete multiple collagens, which increase stiffening and remodel the extracellular matrix.

The fibrotic response comprises multiple stages (Figure 3). Typically, injury of epithelial cells leads to inflammation and initiates fibrosis (42, 46, 56–59). Inflammation recruits mesenchymal-origin cells, mainly fibroblasts, e.g., hepatic stellate cells (HSCs) and alveolar fibroblasts, to the injured parts (42, 46, 56–59). Next, increased expression of fibrosis-driving cytokines, including TGF-β, FGFs, and PDGFs, drives differentiation of fibroblasts to myofibroblasts (42, 46, 60–63) (Figure 3). The TGF-β signaling cascade plays a key role in fibrosis. In healthy tissues, the TGF-β level is minimal. However, upon tissue damage, TGF-β expression increases, which interacts with TGFBRs, resulting in SMAD2 and SMAD3 activation. Next, SMAD2 and SMAD3 heterodimerize with SMAD4 and transcriptionally activate the expression of several profibrotic genes, including collagens (60–63). Additionally, TGF-β induces SMAD-independent pathways to augment fibrotic gene expression (60–63). Altogether, activation of these pathways leads to enhanced production of ECM remodelers (collagens, fibronectins, basement membrane proteins, and α-smooth muscle actin) (40–43, 46). ECM remodeling enhances tissue stiffness, reduces oxygen diffusion (elevating oxidative and hypoxic stress), and eventually compromises organ function and cell death, thereby perpetuating and aggravating the fibrotic damage (40–43, 46–53) (Figure 3).

## Circadian drivers of metabolic liver disease and fibrosis

### The circadian clock and liver physiology.

A healthy liver is critical for maintaining metabolic homeostasis, and the liver CC plays a pivotal role in this process. Studies in mouse livers revealed rhythmic mRNA accumulation for approximately 10%–15% of the genome (13, 21, 28, 29, 64). This rhythmicity in hepatic gene expression largely arises from circadian phase–specific DNA binding of CC genes and clock-regulated transcription factors (13, 64–69). Hepatic circadian transcription involves changes in three-dimensional genome and has implicated a role for REV-ERBα (70–72). Hepatic gene expression is also controlled by zonation, or spatial positioning relative to the liver’s central vein and peripheral portal tracts (73). A combination of single-cell RNA sequencing with FISH revealed that several key metabolic genes are controlled by both zonation and the liver CC (74). Importantly, in mice with hepatocyte-specific knockout of the CC genes *Rev-erba* and *Rev-erbb*, single-nucleus RNA sequencing revealed that the hepatocytic clock dictates cellular communication in liver by regulating gene expression in non-parenchymal cells such as liver endothelial cells and Kupffer cells (75). Interestingly, HSCs have also been shown to control zonation and liver function (76). Lipidomic, proteomic, and metabolomic studies have confirmed the widespread role of the CC oscillator and meal timing in dictating liver physiology (32, 77–81).

Since the role of the CC system in regulating liver metabolism has been reviewed (4, 5, 23, 33–34), here we summarize key features that, when deregulated, contribute to chronic metabolic liver disease resulting in fibrosis (Table 1). The liver CC controls blood glucose levels by regulating both preprandial gluconeogenesis and postprandial glycogen synthesis (4, 5, 23, 82). The liver CC controls glucose metabolism by regulating expression of key genes, e.g., glucokinase (*Gck*), phosphoenolpyruvate carboxykinase (*Pck1*), and glucose transporter 2 (*Glut2*) (4, 5, 23, 82). The CC also influences the glucoregulatory transcriptional activity of CREB, CHREBP, and GR (23, 82–84). Regarding lipid metabolism, plasma triglycerides (TGs), free fatty acids (FFAs), and cholesterol display circadian variations (4, 5, 23, 33, 34) and are disrupted following knockout of CC genes such as *Clock* (85), *Rev-Erba*, and *Rev-Erbb* (86, 87). The liver CC regulates hepatic TG levels by controlling the expression of enzymes such as *Gpat2*, *Lipin1/2*, and *Dgat2*, while REV-ERBα represses *Insig2* and *miR122* levels to regulate SREBP1c, the driver of hepatic de novo lipogenesis (4, 5, 23, 88). The metabolism of FFAs, bile acids, and xenobiotics is also controlled by the CC (4, 5, 23). The liver CC also controls cellular processes such as endoplasmic reticulum (ER) stress, unfolded protein response (UPR), autophagy, and response to reactive oxygen species (ROS), all of which are indispensable for metabolic homeostasis (4, 5, 82, 89–91) (Figure 1).

Recent studies also investigated the CC in myofibroblasts. Using enriched murine HSCs and human myofibroblasts, the existence of CC genes and their circadian expression were demonstrated (92). Furthermore, this study identified nearly 2,000 rhythmically expressed genes involved in metabolism, stress response, collagen/ECM synthesis, and cell cycle in HSCs (92). Among the rhythmically expressed HSC genes were members of the TGF-β/BMP/activin pathways, including *Smad3*, *Smad7*, *Smad6*, receptors (*Acvr1*, *Bmpr1a*, *Tgfbr1*), and ligands (*Bmp2*, *Bmp3*, *Tgfb1*) (92). Future studies (e.g., applying fibroblast-CC mutant mouse models) will be essential to decipher the importance of dynamic circadian communication between different liver cell types in health and disease.

Although the circadian biology of the mouse liver is well studied, the identity of rhythmic genes in human hepatocytes is mostly unknown owing to challenges associated with collecting multiple biopsies over 24 hours. Recent studies (93, 94) performed 24-hour transcriptomic analyses using humanized liver chimeric mice (HLCM) as a surrogate for human liver (95, 96). These investigations unraveled the human hepatocytic circadian transcriptome, including common and distinct rhythmic genes and biochemical processes. These studies also revealed rhythmic pathways shared and discordant between human and mouse hepatocytes (93, 94). Notably, genes metabolizing carbohydrates, FFAs, TGs, and bile acids were shown to display rhythmicity in both human and mouse hepatocytes (94).

### Disruption of the liver clock, metabolism, and fibrosis.

Lifestyle changes and effective antiviral therapies have largely shifted the major causes of liver disease from viral to metabolic diseases. Owing to the global epidemic of obesity, metabolic dysfunction–associated steatotic liver disease (MASLD; formerly NAFLD) is currently emerging as the most prevalent chronic liver disease (CLD), affecting 20%–25% of the world population (97–100). MASLD is the liver manifestation of metabolic syndrome. MASLD encompasses a wide range of conditions, from benign metabolic dysfunction–associated steatotic liver (MASL) to metabolic dysfunction–associated steatohepatitis (MASH), a risk factor for developing hepatocellular carcinoma (HCC) (97–100). Modern diets characterized by overconsumption of energy-dense foods and fructose-containing drinks are key for driving metabolic syndrome. Both systemic and tissue-specific metabolic deregulation leads to hepatic stress and cell death, thereby creating an inflammatory milieu that eventually initiates fibrotic responses (97–100) (Figure 2).

Fibrosis is the major determinant of clinical outcomes in MASH and is associated with increased risk of cirrhosis and HCC (98, 99). In the healthy liver, HSCs are largely non-proliferative. However, liver injury leads to activation of HSCs, driving their differentiation to myofibroblasts, which are proliferative and contractile and upregulate expression of α-smooth muscle actin (αSMA) and multiple collagens. HSC activation results from several signals, such as proinflammatory cytokines, apoptotic hepatocytes, and increased ROS production (48–50, 57, 58). Activated HSCs increase the secretion of profibrotic molecules (TGF-β and PDGF-β), which enable further recruitment of immune cells (48–50, 57, 58). Thus, CLD-induced HSC activation eventually disrupts liver architecture and functions.

Interestingly, the perturbation of the crosstalk between metabolism and CC drives different liver pathologies, including MASLD. MASLD arises due to energy surpluses created by (a) alterations in glucose and FFA metabolism, (b) increased de novo lipogenesis, and (c) compromised β-oxidation or reduced hepatic TG exports (100). TG accumulation in hepatocytes increases ER stress, UPR, and ROS levels, which together act as triggers for MASLD and its sequela, fibrosis, by recruiting immune cells (100, 101). Importantly, metabolic perturbations can alter the functioning of both the hepatic and myofibroblast CC oscillators (5, 33, 34, 92). Circadian misalignment driven by daytime-restricted feeding or jet lag drives liver CC perturbation (102, 103). Meal timing also affects liver proteome, phosphoproteome, and lipidome (32). MASLD features, including increased TG levels and fibrosis, have been noted in mice fed with different versions of high-fat diet as well as with changes in meal timing (23, 27, 32, 34, 35). MASLD breaks down the HSC CC oscillator, and this correlates with elevated expression of profibrotic collagens and αSMA (92) (Table 1). Furthermore, disruption of CC functioning induced by genetic mutations of *Clock* and *Rev-Erb*s leads to liver steatosis (85–87), while absence of *Bmal1* in hepatocytes is known to predispose to HCC (104). Finally, not only metabolic but also infectious diseases perturb the rhythmic transcriptome in human hepatocytes, and predispose to CLD, fibrosis, and HCC, as shown for chronic hepatitis C virus infection (94).

## Circadian disruption in chronic kidney disease and fibrosis

### Circadian clock and renal physiology.

Kidneys filter blood and maintain fluid and electrolyte balance, which contributes to sustaining blood pressure (BP) (105–111). Circadian variation in renal functions has been observed in several species (112–116), including the circadian rhythmicity of urinary excretion of sodium, potassium, chloride, and phosphate (117, 118). In humans, urinary volume and pH also display 24-hour rhythmicity under relatively constant conditions of eating, drinking, and sleeping (119). Circadian rhythms also exist for the glomerular filtration rate (GFR), corticomedullary osmotic changes, blood flow, and transport of water and electrolytes, which are believed to be driven in part by the kidney CC (105–110). Global disruption of *Bmal1* in rats revealed a sex-dependent dissociation between circadian BP variation and control of sodium excretion, as only female *Bmal1^–/–^* rats had significantly greater sodium excretion during the active phase compared with controls (120). Additionally, murine kidney-specific cadherin-Cre–mediated (*Ksp*-Cre–mediated) *Bmal1* knockout in distal tubules suggests its role in BP control and Na^+^ handling in response to a K^+^-depleted diet, but only in male mice (121).

Kidneys and their functional units, nephrons, comprise over 30 different cell types that contribute to renal physiology. The existence of a “kidney clock” has been noted in rats and mice during embryonic development (122–124). Analyses from mouse fetal kidney (E18–E20) detected thousands of rhythmic transcripts, which included cell cycle and DNA repair genes, drivers of nephron development (*Hoxb7* and *Pax2*), epithelial sodium channel α subunit (*Scnn1a*, encoding αENaC), and sodium/hydrogen exchanger (*Slc9a3*) (105–110). Investigations using global-*Bmal1-*KO and ureteric bud–specific (*Hoxb7* Cre-*Bmal1*-KO) mutant mice have revealed the role of CC in controlling nephron development. RNA sequencing from kidneys of adult mice indicated that approximately 13% of the genome (second only to what is observed in liver) is expressed in a circadian manner, suggesting a crucial role in driving renal physiology. Transcriptomic studies from different kidney parts, e.g., distal convoluted tubule, connecting tubule, and cortical collecting duct, not only revealed cell-intrinsic rhythmic expression of key CC genes but also identified numerous genes (involved in water and electrolyte balance, BP, and metabolic processes) that display circadian expression and were disrupted in *Clock*-KO mice (105–110, 115, 125). Aldosterone controls expression of *Scnn1a*, which plays a major role in sodium handling and BP regulation (106–109). The CC gene *Per1* participates in the aldosterone-mediated expression of *Scnn1a*, which further links renal CC machinery and a mediator of sodium balance (126).

Posttranscriptional mechanisms also contribute to the rhythmic gene expression in the kidney (127). Ribosome profiling from mouse kidney revealed a circadian translation pattern for several genes with known roles in renal functions, including aquaporins (*Aqp2*, *Aqp4*, *Aqp8*), podocin (*Nphs2*), the enzyme *Cyp24a1*, transporters (*Glut9*, *Pept1*), adenosine receptor (*Adora1*), and *Ppara* (127). The circadian rhythm of GFR (independent of cardiac function and autonomic nervous system) is critical for maintaining BP. Consistently, podocyte-specific (*Nephrin* Cre-driven) *Bmal1* knockout led to perturbed diurnal GFR and BP (128, 129), raising novel pathophysiological questions about the relationship between podocytes and glomerular hemodynamics. Several renal-derived hormones (angiotensin, endothelins, and aldosterone) display rhythmic production/degradation, and the CC genes *Cry1/2* and *Per1* are known to participate in this regulation (129–131). Collectively, these investigations confirm that key features of renal physiology (such as BP and electrolyte control and hormone production) display circadian rhythmicity in both humans and mice (132) (Figure 1).

### Circadian clock disruption in kidney disease and fibrosis.

Chronic kidney disease (CKD) is a global health problem affecting approximately 10% of the population that increases the risks of morbidity and mortality (133–136). CKD may originate from several heterogeneous pathological conditions that damage the cellular structures of kidney, leading to permanent loss of function. The pathogenesis of CKD and renal fibrosis has been extensively reviewed (51, 52, 133, 134). Briefly, sustained pathological insults stemming from multiple conditions (metabolic syndrome/diabetes, drug toxicity, cardiovascular diseases, autoimmunity) lead to immune cell infiltration in the interstitial and glomerular regions (46, 51, 52). This inflammatory response is largely driven through the activation of NF-κB and MAPKs (p38 and JNK), leading to increased production of several pathogenic cytokines, chemokines, and growth factors (46, 51, 52). Persistence of this proinflammatory environment drives activation of pericytes as well as differentiation of myofibroblasts and phenotypic changes characterizing the epithelial-mesenchymal transition, which, along with TGF-β–induced deposition of collagens and fibronectin in the ECM, results in renal fibrosis (46, 51, 52, 133–136). In contrast to liver and lungs, the functional role of the kidney fibroblast–specific CC in health and disease remains unknown (Table 1).

CKD is associated with perturbed diurnal rhythmicity of BP, i.e., a non-dipping pattern, which is independently correlated with a higher mortality rate or predisposition toward end-stage renal disease (105–110, 135). Polymorphisms in the human *BMAL1* gene are associated with hypertension and type 2 diabetes (137), both of which predispose to CKD. CKD disrupts sleep patterns (138, 139), which are also documented in animal models of kidney disease (partial nephrectomy, adenine-induced) (105–110). The renin-angiotensin-aldosterone system is altered in CKD, and *Bmal1*-KO mice display perturbations in renin production, GFR, and BP (140). Thus, excess renin production may contribute to high BP and cardiorenal fibrosis. Another potent profibrotic mediator, the hormone endothelin-1 (ET-1), is a *Per1* target gene expressed in collecting duct cells and in the renal cortex (105–108).

Animal models have indicated that CC perturbation predisposes toward renal fibrosis. Notably, genomic-*Bmal1*-KO mice show aggravated unilateral ureteral obstruction–induced (UUO-induced) interstitial fibrosis (140). Moreover, owing to increased TGF-β activity and *Cox2* expression, *Clock*-KO mice develop more severe renal fibrosis upon ureteral obstruction compared with control mice (141). Whether kidney fibroblast–restricted CC gene mutations aggravate renal fibrosis remains unknown. Deoxycorticosterone acetate–treated (DOCA-treated) mice are a clinically relevant model for developing renal inflammation and fibrosis (142). DOCA alters the renal expression of CC genes, suggesting a link between behavior-induced (diet-induced) alteration of clock and CKD (142). Notably, BMAL1 regulates the circadian expression of *Nrf2*, a master regulator of antioxidant responses protecting the kidney (143). The NRF2 target genes *Hmox1* and *Pparg* mediate glomerular protection in experimental diabetic glomerulopathy (144) and immune-mediated crescentic glomerulonephritis, respectively (145). Collectively, these investigations suggest that disruption of renal CC function, which normally controls renal physiology, may drive CKD and renal fibrosis. Future studies will be required to unravel the role of kidney cell type–specific (epithelial, fibroblast, and immune) CC mutants in CKD and renal fibrosis (Table 1).

Clock disruptions predispose to chronic respiratory disease and lung fibrosis.

### Circadian biology of pulmonary functions.

Various aspects of respiratory activity are known to display a 24-hour variation in various species (146–150). Sleep-wake cycle influences the daily rhythmicity of pulmonary ventilation (*V_E_*). The metabolic rate drops during sleep and accompanies a decrease in *V_E_*. In healthy humans, several aspects of lung function, e.g., forced expiratory volume in 1 second (FEV1), peak expiratory flow (PEF), and airway resistance, display diurnal behavior (146–151). In controlled settings, it was observed that healthy human lungs attain their peak functional capacity (determined by FEV1 and FEV1/FVC [forced vital capacity]) around noon (mid-active phase), which gradually decreases and reaches its lowest level at midnight/early morning (late-rest phase) (151, 152). Recordings of gaseous metabolism and body temperature in rats under controlled conditions have also confirmed the circadian rhythmicity in *V_E_*, which closely matches the pattern displayed by the *VO_2_* and *VCO_2_* (measurements of oxygen consumed and carbon dioxide exhaled, respectively, in milliliters per minute). Rhythmicity in *V_E_* was also noted in other species, suggesting an evolutionarily conserved phenotype (146). Additionally, in mouse lungs diurnal variations exist for recruitment of immune cells (B cell, granulocytes, and macrophages) (150). These studies indicate that the circadian system contributes to the daily rhythmicity observed for essential pulmonary functions in both humans and mice.

Molecular drivers and successive stages of lung development are considerably similar in mice and humans (153). In utero investigations in mice and rats first detected expression of CC genes at a time frame roughly correlating with the pseudoglandular stage of lung development (E12–E17 in mice, corresponding to 5–17 weeks after conception for humans) (153, 154). A histological study of human and rabbit bronchioles led to the identification of Clara cells (now termed club cells), which play essential roles in maintaining lung homeostasis (153). The transcriptional activity of NKX2.1 (TTF1) is key for lung development and production of surfactant protein A from the Clara cells (153–160). Importantly, *TTF1* expression correlates with the transcription of *Cry2* and *Clock* during lung development in mice. In prenatal murine lung, *Rev-Erba* is presumed to regulate oxidative and inflammatory stress (149). Investigations using *Per2*-luciferase mice provided the initial evidence for suprachiasmatic nucleus–entrained rhythmic clock in the mature lungs (154). CC genes are expressed in mouse larynx, trachea, bronchus, and lungs (161). Importantly, circadian expression of CC genes and several of the muscarinic acetylcholine receptor genes (*Chm2*, *Chm3*, and *Chm4*) in respiratory tissues of mice was lost following double knockout of *Cry1* and *Cry2* (*Cry1^–/–^Cry2^–/–^*) (161). Importantly, circadian disruptions modeling shiftwork and jet lag are known to disrupt CC gene expression and lung function (162). Functional CC oscillators are known to be present in different lung cell types, e.g., alveolar and bronchial epithelium (club cells), lung fibroblasts, and macrophages (163–166) (Table 1). Critically, club cells are known to maintain CC coherence among various cell types of lungs, as specific ablation of these cells leads to altered rhythmicity in the remaining tissue (166). Transcriptomic analyses from mouse lungs identified approximately 1,000 genes that are expressed in a diurnal manner, and pathway analyses indicated that the majority are immune related (166). The role of the CC oscillator in pulmonary airway epithelial cells (AECs) was addressed by selective *Bmal1* knockout in club cells. RNA sequencing showed that the AEC CC controls expression of genes involved in metabolism of lipids and xenobiotics, ECM remodeling, and chemokine/cytokine signaling (166). Microarray-based study in rats identified numerous genes displaying circadian expression patterns, of which nearly 60% were expressed in the rest (inactive) phase (167). Among the cycling transcripts in rat lungs are genes implicated in maintenance and repair of lung parenchyma, and vasculature (167). Collectively, these investigations have established the “clock” as a major regulator of respiratory functions.

### Clock connection to chronic respiratory disease and fibrosis.

Chronic respiratory disease is exemplified by chronic obstructive pulmonary disease (COPD) and asthma, which both represent global health concerns affecting quality of life and mortality. Circadian biology influences various aspects of lung disease and fibrosis (53, 54, 146–150, 168). COPD and asthma symptoms worsen in the early morning. A study involving more than 7,000 asthmatic patients reported that around 60% suffered from nighttime symptoms more than three times each week, while 40% suffered every night (168). Multiple factors, including oxidative stress, mucus production/levels, lung inflammation, and cortisol levels, influence the nighttime severity of asthma (168–170). Allergen-driven hyperactivity of eosinophils and mast cells drives bronchospasm. In humans and mice, mast cells and eosinophils are known to harbor functional CCs that regulate the expression of several disease-related genes (171, 172). Notably, IgE-mediated activation of mast cells and consequent release of histamines and leukotrienes were found to exhibit circadian rhythmicity, and *Bmal1* and *Rev-Erba* transcripts were reduced in bronchioles of asthmatic mice (173). In allergic asthma models, macrophage-specific *Bmal1*-KO mice show increased lung inflammation associated with increased IL-5 levels (173). Furthermore, club cell–specific *Bmal1* knockout is known to drive hyperinflammatory responses upon LPS challenge and bacterial infection as a result of perturbed diurnal expression of *Cxcl5* (164). Critically, inflammation can disrupt the expression of CC genes in lungs and reprogram the transcriptome and metabolome (146, 147). Collectively, these results suggest that CC perturbation in various cell types of lungs predisposes to inflammatory airway diseases like asthma.

COPD is a chronic disease resulting in reduction of respiratory function. The severity of COPD symptoms in humans shows marked diurnal variation (worsening during late night/early morning) (174, 175). COPD results in oxygen desaturation, thus worsening sleep quality and heightening the risk of mortality due to cardiovascular pathologies (174, 175). A cross-sectional study noted abnormal sleep patterns in approximately 70% of COPD patients, which suggests a feedforward cycle in which sleep disturbance and COPD worsen each other. Chronic exposure to cigarette smoke (CS) is a major cause of COPD (176, 177). In mouse models of COPD, CS altered the expression of *Rev-Erba* and *Per2*. Importantly, reduction of REV-ERBα protein levels was observed in COPD patients (146). Genetic studies support a role for REV-ERBα activity in reducing overall inflammation (decreased neutrophil levels and cytokine expression) in COPD (178, 179). Additionally, in mouse lungs, CS-induced COPD reduced levels of *Sirt1* (179), a key regulator of CC. Decreases in SIRT1 activity were found in COPD patients (180). Alteration of molecular CC functioning has been linked to pulmonary fibrosis (181–184). Investigations using the *Clock^Δ19^* model showed that the mutants spontaneously develop a fibrotic phenotype with an increase in ECM remodeling gene expression, correlating with higher collagen deposition around bronchioles (181). Mechanistically, CLOCK DNA binding was found to regulate the expression of *Nrf2*, a regulator of ROS signaling (181). And *Clock^Δ19^* mice presented with reduced *Nrf2* and increased oxidative damage (181). REV-ERBα activation could prevent progression of pulmonary fibrosis by limiting TGF-β activity (182, 183). Finally, age-associated lung fibrosis is increased in *Bmal1*-KO mice (184). Collectively, these investigations reveal that perturbation of the CC is causally linked to chronic respiratory disease and lung fibrosis.

## Clinical impact: chronotherapeutic basis for targeting diseases

The simultaneous deployment of genetics and various genome-wide approaches in model systems has revealed the widespread role of the clock machinery in maintaining physiology and thus potentially affecting various chronic diseases including fibrosis. Translating these findings to novel therapeutic opportunities will require the integration of different aspects of circadian biology into clinical practice (185–188). Chronopharmacology studies circadian variation to determine optimal timing for drug administration. Indeed, reduced drug toxicity and increased therapeutic efficacy of the colorectal cancer drug oxaliplatin are observed when it is administered in late afternoon versus early morning (189). Time-dependence of treatment response and survival was observed in two recent studies on immunotherapy (190, 191). Moreover, many therapeutic drugs target proteins encoded by genes that are expressed in a circadian manner (192). Thus, chronotherapy holds vast promise for various diseases (185–187).

CC is a major regulator of metabolism. Behavioral modifications, such as restriction of eating to the active phase (in the absence of “binge” eating), improvement of sleep quality, and minimizing of light exposure in rest period, have been shown to improve metabolic homeostasis. Critically, time-restricted eating has been shown to significantly improve obesity, glucose levels, cardiovascular disease, MASH, and liver fibrosis (193–195).

Interestingly, several MASH therapeutic targets are regulated by the CC (4, 5, 23). Indeed, the CC regulates (a) FXR and bile acid metabolism (targeted by FXR agonists), (b) FGF-21 (targeted by antifibrotic compounds in clinical development), and (c) PPAR, the target of elafibranor to treat primary biliary cholangitis. Resmetirom, the first FDA-approved drug for MASH, targets thyroid hormone receptor β to improve mitochondrial fatty acid oxidation and reduce intrahepatic lipid accumulation (196, 197).

In mouse models of metabolic syndrome and MASLD, agonists of CRY (KL001) and REV-ERBs (SR9009 and SR9011) have been shown to improve disease parameters (obesity, glucose tolerance, and lipid profiles) (23, 27). SR9009 reduced expression of profibrotic genes and inflammatory genes, which led to reduction in features of liver fibrosis (92). However, it should be noted that SR9009 displayed REV-ERB–dependent and –independent effects on gene expression. Additionally, REV-ERB activation has been shown to significantly improve lung fibrosis: In different models of lung fibrosis, pharmacological activation of REV-ERB prevented overexpression of collagens and lysyl oxidases (182, 183). Critically, in organotypic cultures from idiopathic pulmonary fibrosis (IPF)patients, REV-ERB agonists prevented the activation of myofibroblasts to IPF-driving fibroblasts that secrete collagen (182, 183). Notably, existing treatments for asthma and COPD (prednisolone and steroid) show circadian variations in their efficacy to reduce inflammation (198). With respect to CKD, multiple observational studies have confirmed that chronotherapeutic methods can improve efficacy of drugs, including furosemide, thiazides, valsartan, and antihypertensive drugs (185–187).

Collectively, the integration of circadian biology into clinical medicine provides novel opportunities to improve the prevention and treatment of chronic diseases and fibrosis. Detailed mechanistic studies as well as the investigation of cell-specific treatment approaches will be required to fully harness CC biology for therapeutic discovery and clinical management.

## Knowledge gaps and future perspectives

**Figure 4:**
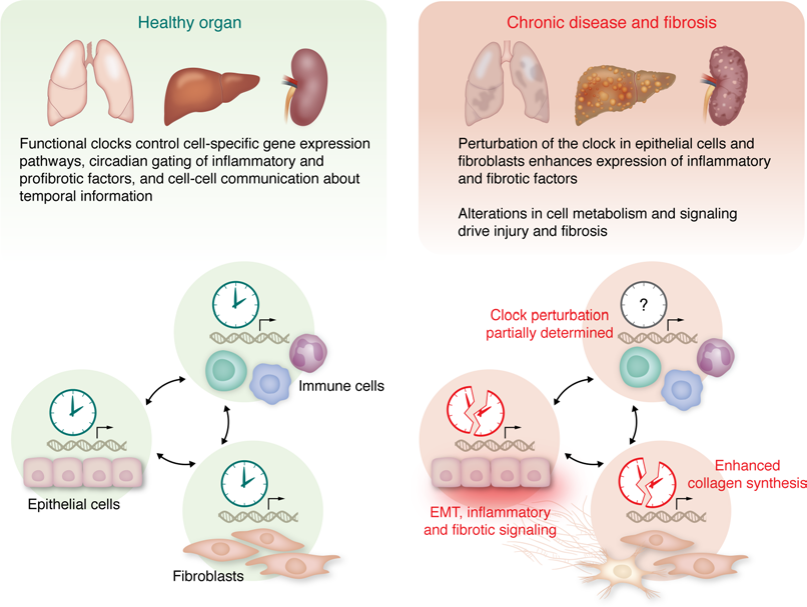
Future perspectives and knowledge gaps. In a healthy organ (left), CC-controlled functions maintain homeostasis by driving essential gene expression programs. Emerging evidence suggests that the different CCs also dictate communication between different cell types and organs to maintain physiology. Importantly, perturbation of the CC oscillator function in different cell types alters the rhythmic gene expression programs and drives toward chronic diseases involving fibrosis (right). Although scientific advances have revealed some of the fundamental concepts of circadian biology, several crucial events pertaining to both health and diseased states still remain to be determined (Table 2).

Despite considerable progress in recent years, several knowledge gaps exist (Figure 4 and Table 2). Addressing these gaps will provide opportunities for a better understanding of the impact of CC on disease biology and fibrosis as well as therapeutic discovery. Organs likely crosstalk in a temporally specified (circadian) manner, as this helps to establish synergy between organ-specific gene expression programs and biochemical reactions and to maintain homeostasis. An example of this complexity is the maintenance of glucose homeostasis requiring CC-dictated crosstalk between (a) liver (gluconeogenesis and glycogen synthesis), (b) pancreas (insulin and glucagon production), (c) intestinal release of GLP1 (an approved drug target for obesity), and (d) glucose uptake in skeletal muscle.

While for some cell types, such as liver and lung epithelial cells or myofibroblasts, common concepts in CC perturbation and disease biology have been described (Table 1), the overarching mechanisms that dictate how various organs crosstalk during chronic disease in a diurnal manner remain largely unknown (Figure 4 and Table 2). The understanding of these mechanisms will provide clues to organ-specific disruption of gene networks. Thus, future investigations should explore at multiple levels (transcriptome, proteome, metabolome) the signals, ligands, and receptors that enable organs to talk to each other, revealing intra- and extracellular maps of communication.

Furthermore, the development of chronic disease and fibrosis in an organ involves interactions between several cell types (epithelial, fibroblasts, and immune cells) with extensive intra- and intercellular crosstalk, influencing disease outcomes. An example is circadian changes in actin polymerization, shown to drive fibroblast mobilization during skin wound healing (199). However, at present the circadian interactions between different cell types in health and disease are largely unknown (Figure 4 and Table 2). Thus, it will be essential to explore, e.g., at the single-cell level, the circadian expression of CC-controlled gene networks that dictate interactions between epithelial, fibroblast, and immune cells.

Additionally, whether polymorphisms in key CC genes, e.g., *BMAL1*, predispose to chronic diseases and fibrosis of lungs, liver, or other organs remains poorly understood. Furthermore, it will be essential to employ emerging advanced modeling approaches including artificial intelligence and machine learning to integrate data from patients and model organisms to (a) mechanistically understand the common and unique ways of circadian gene regulation in different tissues, and (b) predict the outcome of drug treatments. The understanding of these mechanisms may ultimately lead to discovery of novel targets and drugs.

While there are many mechanistic investigations of circadian biology in mouse models, knowledge from human or patient tissues is rare, which limits the understanding of potential clinical translation. Mice are nocturnal, while human beings are diurnal. Also, murine experimentation is conducted under uniform laboratory conditions (feeding, temperature, humidity, and “light” duration, etc.), which are not observed in day-to-day activity of humans. Furthermore, with respect to CC genes, widely documented variations between individuals confer distinct chronotypes, a term describing human behavioral phenotypes that influence the “timing” of sleep-wake and rest-active periods; these chronotypes are very likely to influence individual disease biology or drug responses. Moreover, how different cell types respond to disease conditions in a circadian manner remains poorly understood. Furthermore, direct targeting of ubiquitously expressed CC genes over the long term may affect multiple organs and have undesirable side effects, which will require additional studies.

Collectively, a detailed investigation of CC oscillator activity and CC-regulated cell circuits in different cell types and organs combined with patient studies for clinical translation will unravel novel approaches to halt progression of chronic disease and prevent or treat fibrosis.

## Figures and Tables

**Table 1:**
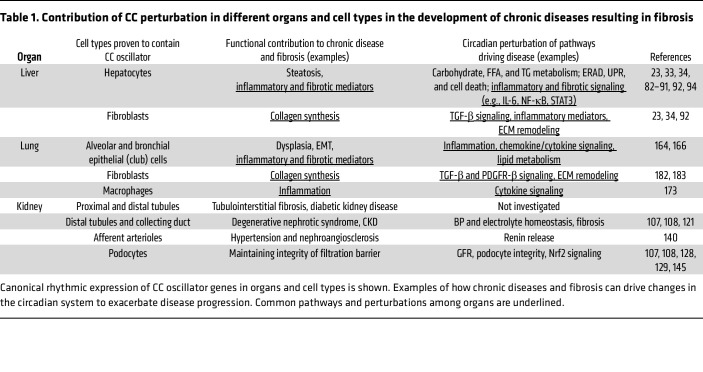
Contribution of CC perturbation in different organs and cell types in the development of chronic diseases resulting in fibrosis

**Table 2:**
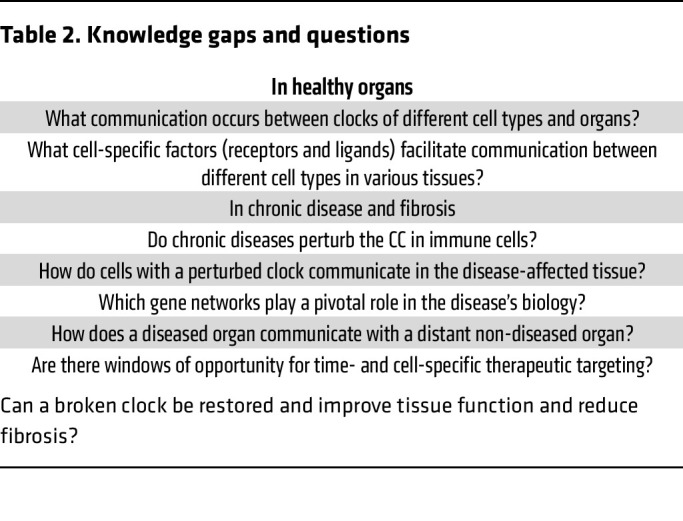
Knowledge gaps and questions

## Version 1 10/01/2025

Electronic publication
